# Immune microenvironment in intervertebral disc degeneration: pathophysiology and therapeutic potential

**DOI:** 10.3389/fimmu.2025.1563635

**Published:** 2025-07-04

**Authors:** Qian Ren, Ling Chen, Yibo Ma, Yansheng Huang, Sibo Wang

**Affiliations:** ^1^ Department of Joint Surgery, Honghui Hospital, Xi'an Jiaotong University, Xi’an, Shanxi, China; ^2^ Department of Spine Surgery, First Affiliated Hospital of Shihezi University, Shihezi, Xinjiang, China; ^3^ Department of Urinary Surgery, Xi ‘an Jiaotong University Medical College Affiliated City Ninth Hospital, Xi’an, Shanxi, China; ^4^ Department of Spine Surgery, Honghui Hospital, Xi'an Jiaotong University, Xi’an, Shanxi, China

**Keywords:** intervertebral disc degeneration, immune microenvironment, immune modulation, immune cells, therapeutic strategies

## Abstract

Intervertebral disc degeneration (IDD) is a prevalent and debilitating condition that affects millions worldwide, leading to chronic back pain and a reduced quality of life. This review shifts the focus to the pivotal role of the immune microenvironment in IDD, highlighting its dual functions—exacerbating degeneration through chronic inflammation while also offering protective mechanisms under certain conditions. Recent research highlights how immune cells such as macrophages, T cells, and B cells, along with cytokines like IL-1β, TNF-α, and IL-6, play dual roles in both exacerbating and potentially mitigating disc degeneration. Key signaling pathways, including NF-κB, MAPK, JAK-STAT, and the NLRP3 inflammasome, are discussed to illustrate their involvement in disc cell apoptosis, extracellular matrix degradation, and chronic inflammation. By synthesizing current research, this review underscores the potential of novel therapeutic strategies that target immune modulation. Anti-inflammatory drugs, biologics, stem cell therapy, and gene editing technologies are explored as promising avenues for treatment. Understanding the immune landscape of IDD not only enhances our knowledge of its pathogenesis but also opens new possibilities for effective, targeted therapies, aiming to improve patient outcomes and reduce the societal burden of this debilitating condition.

## 1 Introduction

Intervertebral disc degeneration (IDD) is a prevalent and far-reaching spinal disorder characterized by progressive degeneration of disc structure and function. The normal intervertebral disc consists of the annulus fibrosus, nucleus pulposus, and cartilaginous endplates, and its primary function is to absorb and distribute mechanical loads, thereby maintaining flexibility and stability of the spine. With age, the water content and elasticity of the intervertebral disc decreases, resulting in impairment of its load-carrying and distribution functions (1). This degenerative process involves multiple pathophysiologic mechanisms, including extracellular matrix (ECM) degradation, apoptosis, and inflammatory responses (2). Key enzymes, such as matrix metalloproteinases (MMPs) and deintegrin metalloproteinases bound to the structural domain of thrombopoietin (ADAMTS), play an important role in ECM degradation, destroying collagen and proteoglycans, thereby compromising the structural integrity of the disc. In addition, oxidative stress and nutritional deficiencies induce apoptosis and autophagy in intervertebral disc cells, further impairing their repair capacity (3, 4). Clinically, IDD mainly manifests as chronic lower back pain and sciatica, with symptoms that may be continuous or intermittent and are often accompanied by radiating pain and sensory abnormalities in the lower extremities. Severe herniated discs may also lead to spinal instability and limited mobility, significantly affecting the patient’s daily life and ability to work. (5).

The prevalence of IDD is high worldwide and is the leading cause of chronic lower back pain. Statistics show that approximately 80% of the population will experience lower back pain at least once in their lifetime, and the prevalence of IDD among people over the age of 40 reaches 60-80%. In the United States, IDD-related lower back pain is the leading cause of work absenteeism and decreased productivity, resulting in tens of billions of dollars in medical costs and indirect economic losses each year. (6). Therefore, elucidating the pathophysiological mechanisms of IDD and developing effective treatment strategies are crucial for improving patient quality of life and alleviating public health burdens. Investigating the immune microenvironment in IDD could unveil novel therapeutic targets, providing new insights and approaches for the prevention and treatment of this debilitating condition.

IDD has long been considered an irreversible degenerative disease primarily driven by age-related physiological changes (7, 8). Traditional treatments have focused on symptom relief and improving quality of life, including conservative and surgical treatments. Among conservative treatments, physical therapy, nonsteroidal anti-inflammatory drugs (NSAIDs), steroid injections, and lifestyle modifications can provide some pain relief, but cannot fundamentally reverse or stop disc degeneration (9). Surgical treatments (e.g., discectomy, spinal fusion, and artificial disc replacement), on the other hand, are aimed at severe degeneration cases, but these methods also have certain risks and limitations (10, 11).

The immune microenvironment plays a crucial role in maintaining tissue health and regulating disease processes, and includes a variety of immune cells (e.g., macrophages, T cells, and B cells) and secreted cytokines and chemokines. Under normal conditions, the immune microenvironment maintains tissue homeostasis by removing cellular debris, fighting pathogens, and promoting tissue repair. However, under pathological conditions, the immune microenvironment may undergo aberrant changes leading to chronic inflammation and tissue damage (12). In recent years, more and more studies have begun to focus on the role of the immune microenvironment in IDD, revealing the dual role of the immune response in the degenerative process. (13, 14) On the one hand, immune cells and inflammatory cytokines can play a protective role by removing degenerating cells and tissue debris, regulating ECM degradation, and promoting repair processes (15). On the other hand, excessive or sustained immune responses can lead to chronic inflammation, accelerated ECM degradation and apoptosis, and thus accelerated disc degeneration (16, 17). Specifically, the role of macrophages in IDD has garnered significant attention. Macrophages can polarize into functionally distinct M1 (pro-inflammatory) and M2 (anti-inflammatory) types (18). Studies have found that in the early stages of IDD, the number of M1 macrophages increases, secreting large amounts of pro-inflammatory cytokines (such as IL-1β and TNF-α), which not only directly damage the ECM but also activate other immune cells, further exacerbating the inflammatory response (19). In the repair stage, M2 macrophages promote ECM repair and regeneration by secreting anti-inflammatory cytokines and growth factors (such as IL-10 and TGF-β) (20). Additionally, the application of new technologies such as single-cell RNA sequencing has enabled researchers to more precisely analyze the cellular composition and gene expression profiles within disc tissues, uncovering the specific roles of different immune cell subsets in IDD (21). These research findings have deepened our understanding of the pathophysiological mechanisms of IDD and provided a theoretical foundation for developing novel immunomodulatory therapies. For instance, modulating macrophage polarization to inhibit excessive inflammation or promote tissue repair could be an effective strategy for future IDD treatments (22).

This review aims to systematically summarize the role of the immune system in the process of IDD and explore its therapeutic potential. In recent years, an increasing number of studies have shown that immune cells and inflammatory cytokines play critical roles in IDD, influencing both the progression of degeneration and tissue repair and regeneration. By reviewing the latest research advances, this review aims to reveal the dual role of the immune system in IDD and discuss the feasibility and prospects of innovative immunomodulatory therapeutic strategies.

## 2 Anatomy and immunological characteristics of the intervertebral disc

### 2.1 Structure and function of the intervertebral disc

The intervertebral disc, located between the vertebral bones, is composed of three main parts: the annulus fibrosus, the nucleus pulposus, and the cartilage endplates (23, 24). The annulus fibrosus is a tough outer ring composed of multiple layers of collagen fibers and chondrocytes, primarily functioning to provide structural support and restrict the movement of the nucleus pulposus (25). The nucleus pulposus, located at the center of the disc, contains a high-water content, proteoglycans, and collagen, serving mainly to cushion pressure and distribute loads. The cartilage endplates cover the upper and lower surfaces of the disc, composed of hyaline cartilage, protecting the vertebral bodies and transmitting loads (1). The primary function of the intervertebral disc is to absorb and distribute mechanical pressures generated by daily activities and movements (26). It achieves this through the gel-like properties of the nucleus pulposus and the tough structure of the annulus fibrosus, enabling the spine to flexibly move and maintain stability (27). Additionally, the intervertebral disc plays a crucial role in maintaining the height and shape of the spine, ensuring normal spinal curvature and rotation.

### 2.2 Composition of the immune microenvironment

The immune microenvironment of the intervertebral disc consists of various immune cells, cytokines, and other signaling molecules (28, 29). Under normal conditions, the disc contains very few immune cells due to its low blood supply and immune-privileged status (30). The main immune cells include macrophages, T cells, and B cells (15). Macrophages are key regulators of the disc’s immune response, playing vital roles in tissue repair and the clearance of cell debris (31). T cells and B cells, as components of the adaptive immune system, are crucial in chronic inflammation and immune memory (32). Additionally, neutrophils and natural killer (NK) cells also play important roles in acute and chronic inflammatory responses (33). Cytokines such as IL-1β, TNF-α, and IL-6 are critical in both healthy and pathological states of the disc. These cytokines influence disc function and health by regulating cell migration, inflammatory responses, and tissue repair (4, 34). Chemokines, on the other hand, guide immune cell migration to sites of damage or infection, thereby participating in the regulation of the immune microenvironment (30, 35).

### 2.3 Immunological characteristics of the intervertebral disc

Under normal conditions, the low oxygen environment and lack of vascular supply within the disc restrict the entry of immune cells (36). However, during disc degeneration, this immune privilege can be compromised, leading to immune cell infiltration and chronic inflammation. Common immune cells found in degenerated discs include macrophages, T cells, and neutrophils (37). Studies indicate that the increase in inflammatory cytokines and immune cells in degenerated discs is closely related to disc tissue degradation and pain (7, 38). For instance, the upregulation of cytokines such as IL-1β and TNF-α further enhances the activity of degradative enzymes like MMPs and ADAMTS, leading to ECM breakdown (39). These inflammatory cytokines not only directly damage the disc’s ECM components but also induce cell apoptosis and inhibit cell proliferation, thereby accelerating disc degeneration (40).

Understanding these immunological characteristics and their changes is crucial for developing new therapeutic strategies (41). Research indicates that modulating the immune response within the disc can reduce inflammation and tissue damage, thereby delaying or reversing disc degeneration (42). For example, using anti-inflammatory drugs and biologics can effectively inhibit the activity of key inflammatory cytokines, thereby alleviating pain and improving function (43, 44). Additionally, cell therapy and gene therapy have shown potential in modulating the disc’s immune microenvironment, promising new avenues for future IDD treatments (45). By further studying the immune microenvironment of the intervertebral disc and its changes during degeneration, we can better understand the mechanisms of IDD and explore the potential of immunomodulation in its treatment. This multidisciplinary research approach not only helps to elucidate the pathophysiological mechanisms of IDD but also provides new insights and methods for clinical treatment (43, 46).

## 3 Immune response in the process of intervertebral disc degeneration

### 3.1 Immune cells

IDD is a complex pathological process involving multiple interacting factors, with immune responses playing a crucial role. Various immune cells, such as macrophages, neutrophils, natural killer (NK) cells, and lymphocytes (T cells and B cells), participate in this process, influencing the progression of disc degeneration through different mechanisms (**Table 1**).

**Table 1:** Immune cell types and their roles in IDD.

Immune cell	Role in IDD	Key regulatory molecules	Potential targets and drugs	Clinical implications	References
M1 Macrophages	Secrete IL-1β, TNF-α, and MMPs, promoting inflammation and ECM degradation.	IL-1β, TNF-α, MMPs, NLRP3 inflammasome, ROS, NF-κB, JNK, IL-6, IL-18	TNF-α inhibitors (Etanercept), IL-1β inhibitors (Anakinra), NLRP3 inhibitors (MCC950), ROS scavengers (N-acetylcysteine), NF-κB inhibitors (Parthenolide): IL-6 inhibitors (Tocilizumab)	Targeting M1 macrophages could reduce chronic inflammation and tissue destruction, potentially slowing the progression of IDD. Metabolic reprogramming and combined inhibition of multiple pathways (NF-κB and NLRP3) may offer more comprehensive therapeutic effects.	(31, 48, 49, 174–177)
M2 Macrophages	Secrete IL-10 and TGF-β, promoting tissue repair.	IL-10, TGF-β, Arg-1, CD206, PPARγ, STAT6, IL-4, IL-13, VEGF	IL-10 agonists, TGF-β modulators, PPARγ agonists (Pioglitazone), IL-4/IL-13 cytokine therapy, VEGF modulators	Modulating M2 macrophages could enhance tissue repair and reduce degenerative changes in the disc. Targeting specific pathways like PPARγ and utilizing exosome-based therapies could potentiate M2’s reparative capabilities.	(50, 51, 178–181)
Neutrophils	First responders to injury. Release ROS and proteolytic enzymes, leading to pathogen clearance and tissue damage. Prolonged activation can maintain chronic inflammation.	ROS, Proteolytic enzymes, NETs, CXCL8, MPO, Elastase, MMP-9, IL-17	Antioxidants, Protease inhibitors, NET inhibitors (DNase), IL-17 inhibitors (Secukinumab), MPO inhibitors (AZD5904)	Controlling neutrophil activity could reduce secondary tissue damage and chronic inflammation in IDD. Targeting NETs and neutrophil-derived proteases could mitigate the harmful effects of prolonged neutrophil activation.	(58–60, 182–185)
NK Cells	Kill virus-infected and tumor cells by releasing perforin and granzymes. Interact with macrophages and neutrophils to modulate inflammatory responses.	Perforin, Granzymes, IFN-γ, NKG2D, NKp30, IL-12, IL-15, TGF-β, MICA/B	NK cell modulators, NKG2D antagonists, Checkpoint inhibitors (Anti-PD1), IL-15 superagonists, MICA/B shedding inhibitors, TGF-β inhibitors	Modulating NK cell function might help control excessive immune responses in IDD. NK cell activity can be fine-tuned through the use of checkpoint inhibitors and cytokine therapies, potentially reducing chronic inflammation.	(33, 70–72, 186–188)
T Cells	CD4+ T cells differentiate into Th1, Th2, Th17, and Treg cells. Th1 cells secrete IFN-γ, promoting M1 macrophage activation. Th17 cells secrete IL-17, contributing to chronic inflammation. Treg cells secrete IL-10 and TGF-β, suppressing inflammation.	IFN-γ, IL-17, IL-10, TGF-β, FOXP3, RORγt, STAT3, IL-6, IL-21, PD-1	IL-17 inhibitors (Secukinumab), Treg enhancers, (Low-dose IL-2), PD-1 inhibitors, STAT3 inhibitors, IL-21 inhibitors, FOXP3 gene therapy	Balancing T cell subsets could reduce chronic inflammation and promote tissue repair, offering a potential therapeutic strategy in IDD. Targeting specific cytokines and transcription factors involved in T cell differentiation (e.g., STAT3, FOXP3) could enhance therapeutic outcomes.	(32, 73–76, 78, 151, 189, 190)
B Cells	Secrete antibodies and present antigens. Involved in chronic inflammation and tissue destruction. B cells secrete autoantibodies and cytokines, amplifying immune responses and promoting ECM degradation.	Autoantibodies, Cytokines, CD20, BAFF, APRIL, IL-6, TNF-α	B cell depletion therapy (Rituximab), Cytokine inhibitors (Anti-IL-6, Anti-TNF-α), BAFF/APRIL inhibitors, BTK inhibitors (Ibrutinib), CD20 CAR-T therapy	Reducing B cell activity may help alleviate chronic inflammation and prevent further ECM degradation in IDD. B cell-specific therapies, including BTK inhibitors and CAR-T cells, represent advanced strategies to target B cell-driven pathology in chronic diseases like IDD.	(174–176) (32, 177–181, 191)

IL-1β, Interleukin 1 beta; TNF-α, Tumor Necrosis Factor alpha; MMPs, Matrix Metalloproteinases; NLRP3, NOD-like receptor pyrin domain-containing protein 3; ROS, Reactive Oxygen Species; NF-κB, Nuclear Factor kappa-light-chain-enhancer of activated B cells; JNK, c-Jun N-terminal kinase; IL-6, Interleukin 6; IL-18, Interleukin 18; TGF-β, Transforming Growth Factor beta; Arg-1, Arginase 1; CD206, Cluster of Differentiation 206 (Mannose Receptor); PPARγ, Peroxisome Proliferator-Activated Receptor gamma; STAT6, Signal Transducer and Activator of Transcription 6; IL-4, Interleukin 4; IL-13, Interleukin 13; VEGF, Vascular Endothelial Growth Factor; NETs, Neutrophil Extracellular Traps; CXCL8, C-X-C motif chemokine ligand 8 (also known as IL-8); MPO, Myeloperoxidase; IFN-γ, Interferon gamma; NKG2D, Natural Killer Group 2, member D; NKp30, Natural Killer p30 receptor; MICA/B, MHC class I polypeptide-related sequence A/B; Th1, T helper type 1; Th2, T helper type 2; Th17, T helper type 17; Treg, Regulatory T cells; FOXP3, Forkhead box P3; RORγt, Retinoid-related orphan receptor gamma t; STAT3, Signal Transducer and Activator of Transcription 3; AP-1, Activator Protein 1; CD20, Cluster of Differentiation 20; BAFF, B cell-activating factor; APRIL, A Proliferation-Inducing Ligand; BTK, Bruton’s tyrosine kinase; CAR-T, Chimeric Antigen Receptor T-cell therapy.

#### 3.1.1 Macrophages

Macrophages are among the earliest identified and studied immune cells in IDD. They are widely present in degenerated discs and regulate inflammatory responses and tissue remodeling by secreting various cytokines and enzymes (47). Macrophages play different roles in the early and late stages of disc degeneration. In the early stages, macrophages are primarily of the M1 type, secreting pro-inflammatory cytokines such as IL-1β and TNF-α, promoting inflammation and accelerating ECM degradation (31, 48). These pro-inflammatory macrophages also secrete MMPs, directly damaging the annulus fibrosus and nucleus pulposus structures (49). In the late stages of degeneration, the proportion of M2 macrophages increases. M2 macrophages secrete anti-inflammatory cytokines such as IL-10 and TGF-β, suppressing inflammation and promoting tissue repair (50). However, studies indicate that in chronic degeneration, the imbalance between M1 and M2 macrophages leads to persistent inflammation and tissue damage (51). Recent studies have further revealed the complex role of macrophages in IDD. Zhao et al. (34) found that M1 macrophages promote disc cell apoptosis and ECM degradation through the activation of the NLRP3 inflammasome (52). Additionally, Hou et al. discovered that the polarization state of macrophages can be regulated by specific microenvironmental factors such as hypoxia and high glucose, which are prevalent in degenerated discs (53). Another key finding is the interaction between macrophages and disc cells. *In vitro* co-culture experiments showed that disc cells could attract macrophages by secreting chemokines like CCL2 and regulate macrophage polarization by secreting cytokines such as IL-6 and TNF-α (54). This interaction plays an important role in maintaining the immune microenvironment of the disc. Recent findings have demonstrated that the metabolic features of the degenerated disc—particularly elevated lactate due to hypoxia and increased ROS—are potent modulators of macrophage phenotype (55). Lactate can stabilize HIF-1α and promote M2-like polarization, contributing to a reparative but potentially fibrotic environment (56). Conversely, high ROS levels activate the NF-κB pathway, skewing macrophages toward a pro-inflammatory M1 state (57). These metabolic cues may contribute to the imbalance between M1 and M2 macrophages observed in chronic IDD.

#### 3.1.2 Neutrophils

Neutrophils, as key components of the innate immune system, also play critical roles at different stages of IDD. Neutrophils are among the first immune cells to respond rapidly to injury and infection (58, 59). In the acute stage of disc degeneration, neutrophils are quickly recruited to the lesion site, where they release reactive oxygen species (ROS) and proteolytic enzymes to directly kill pathogens and clear damaged cells (60). However, these molecules can also damage surrounding healthy tissues, further exacerbating disc degeneration (61). In the chronic stage, the persistent presence and activation of neutrophils lead to sustained low-grade inflammation, which is associated with chronic low back pain and further structural damage to the disc (62). Neutrophil migration is primarily regulated by chemokines such as CXCL8 (63). These chemokines are upregulated during disc degeneration, inducing neutrophil aggregation at the lesion site (64). Neutrophils further break down ECM components by releasing various effector molecules such as elastase and MMPs, damaging disc structure. (65). Recent studies indicate that neutrophils not only participate in the initial inflammatory response but also play important roles in chronic inflammation. For example, Dudli et al. (66) found that neutrophils form extracellular traps (NETs) in chronic disc degeneration, which capture and kill invading microbes but also cause self-tissue damage (67, 68). NETs can also interact with macrophages and other immune cells, further exacerbating the inflammatory response. In summary, neutrophils have multifaceted roles in IDD. Initially, they protect by responding rapidly to inflammatory signals, clearing pathogens, and damaged cells. However, the ROS and proteolytic enzymes released by neutrophils can also damage surrounding healthy tissues, especially during chronic inflammation (69). Therefore, careful regulation of neutrophil function is needed in treatment to balance pathogen clearance and tissue protection.

#### 3.1.3 Natural killer cells

NK cells primarily kill virus-infected and tumor cells by releasing perforin and granzymes (70).In IDD, NK cells also participate in regulating local inflammatory responses (71). Although specific studies on NK cells in IDD are limited, there is evidence that NK cells can interact with macrophages and neutrophils to modulate the immune microenvironment in disc degeneration (72).

#### 3.1.4 T cells

T cells play various roles in IDD, including promoting and regulating inflammatory responses (73). CD4+ helper T cells can differentiate into different subsets (such as Th1, Th2, Th17, and Treg cells), each playing distinct roles in inflammation and immune regulation (74). For instance, Th1 cells secrete IFN-γ, promoting M1 macrophage polarization and activation, thus enhancing inflammation. Th17 cells secrete pro-inflammatory cytokines such as IL-17, playing significant roles in autoimmune diseases and chronic inflammation (19). In contrast, Treg cells secrete anti-inflammatory cytokines such as IL-10 and TGF-β, suppressing inflammation and promoting tissue repair (75). The imbalance of T cells in IDD can lead to sustained inflammation and tissue damage (76). Recent studies show that T cells’ roles in IDD extend beyond local inflammatory responses. Weiler et al. (77) found significant T cell infiltration in the disc tissues of IDD patients, with these T cells secreting various cytokines and chemokines to regulate the local immune microenvironment (78). Additionally, T cells can amplify inflammatory responses by interacting with other immune cells such as macrophages and B cells, further exacerbating disc degeneration.

#### 3.1.5 B cells

Despite limited studies on B cells in IDD, their potential roles should not be overlooked. B cells can regulate immune responses by secreting antibodies and presenting antigens (79). Studies show that in autoimmune diseases such as rheumatoid arthritis, B cells promote inflammation and tissue destruction by secreting autoantibodies. In IDD, B cells may participate in chronic inflammation through similar mechanisms. Recent research has further revealed specific roles of B cells in IDD. Significant B cell infiltration was found in degenerated discs, with these B cells promoting local inflammation by secreting autoantibodies and cytokines (80). Moreover, B cells can amplify immune responses by interacting with T cells, leading to further disc tissue damage (81).

#### 3.1.6 Potential therapeutic targets

In chronic stages of IDD, the persistent activation and dysregulation of T cells and B cells lead to chronic inflammation and tissue destruction. Therefore, regulating the functions of T cells and B cells may be a potential strategy for treating IDD. For example, inhibitors targeting specific T cell subsets, such as IL-17A antibodies or Treg cell enhancers, may reduce chronic inflammation and promote tissue repair (82, 83). Clinical trials involving IL-17A inhibitors, like Secukinumab, have demonstrated their potential in treating inflammatory conditions such as ankylosing spondylitis, providing a rationale for their application in IDD (84).

Similarly, Treg cell enhancers, such as low-dose IL-2 therapy, have been explored in early-phase clinical trials for autoimmune diseases, showing promising results in enhancing Treg function and reducing pathological inflammation (85). These findings suggest that modulating Treg cells could be beneficial in IDD by restoring immune balance and promoting tissue repair. For B cells, depletion therapies like Rituximab, which targets CD20+ B cells, have been successful in treating rheumatoid arthritis by reducing B cell-mediated chronic inflammation (86). Although specific studies in IDD are limited, these results imply that B cell depletion could potentially control the chronic inflammation seen in advanced stages of IDD. Additionally, small molecule inhibitors targeting B cell activation pathways, such as Bruton’s tyrosine kinase (BTK) inhibitors, are currently under investigation in clinical trials for various autoimmune conditions, offering another avenue for potential IDD treatment (87).

**Figure 1:**
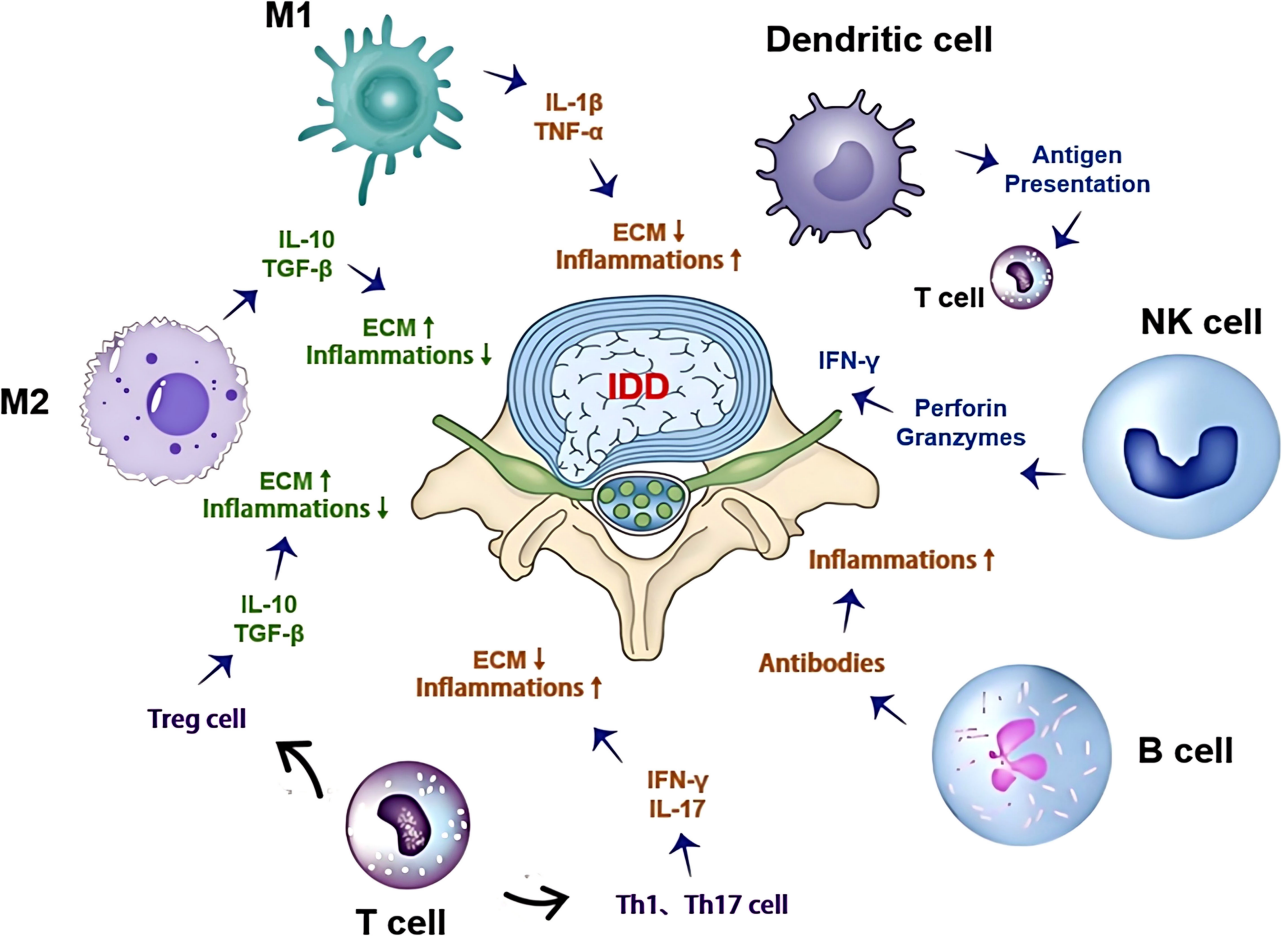
Complex interactions of the immune microenvironment with cell types, signaling pathways, and ECM degradation in intervertebral disc degeneration: This figure illustrates the complex interactions within the immune microenvironment during IDD. It includes various immune cells such as M1/M2 macrophages, T cells, B cells, NK cells, and dendritic cells, highlighting their roles in IDD. These cells secrete multiple cytokines and chemokines, which regulate the degradation and repair processes of the ECM. Arrows indicate the interactions and signaling pathways between these cells, illustrating the dual role of the immune microenvironment in either promoting or inhibiting disc degeneration.

In summary, T cells and B cells play critical roles in the progression of IDD by regulating the local immune microenvironment and amplifying inflammatory responses. Immunomodulatory therapies targeting these cells, supported by evidence from other inflammatory and autoimmune diseases, may represent novel strategies for treating IDD. Future research should focus on validating these approaches in the context of IDD through targeted clinical trials to improve patient outcomes (**Figure 1**).

### 3.2 Cytokines and chemokines

During IDD, inflammatory factors play a crucial role. These factors mediate local inflammatory responses and affect disc cell survival, apoptosis, and matrix degradation (**Table 2**).

**Table 2:** Key cytokines and chemokines in IDD and their potential therapeutic targets.

Cytokine/chemokine	Role in IDD	Key regulatory molecules	Potential targets and drugs	Clinical Implications	References
IL-1β	Key pro-inflammatory cytokine promoting ECM degradation and disc cell apoptosis.	NF-κB, MAPK, Caspase-1, NLRP3 inflammasome	IL-1β inhibitors (Anakinra), NF-κB inhibitors (Parthenolide), Caspase inhibitors (Z-VAD-FMK), NLRP3 inhibitors (MCC950)	Targeting IL-1β can mitigate ECM degradation and reduce disc cell apoptosis, potentially slowing the progression of IDD. Combination therapy targeting both IL-1β and its downstream pathways could offer more comprehensive protection.	(34, 175, 176, 192). (34, 193)
TNF-α	Promotes disc cell apoptosis, ECM degradation, and chronic inflammation.	NF-κB, JNK, AP-1, Caspase-3	TNF-α inhibitors (Etanercept), JNK inhibitors (SP600125), Caspase inhibitors, AP-1 inhibitors	TNF-α is a central mediator of inflammation and cell death in IDD. Inhibition of TNF-α and its associated signaling pathways could reduce chronic inflammation and protect against ECM degradation.	(34, 89, 90, 194, 195)
IL-6	Multifunctional cytokine involved in inflammation and immune response modulation. Plays dual roles in promoting and resolving inflammation.	JAK-STAT3, NF-κB, MAPK	IL-6 inhibitors (Tocilizumab), JAK inhibitors (Tofacitinib), STAT3 inhibitors, MAPK inhibitors (U0126)	Targeting IL-6 and it’s signaling pathways could reduce chronic inflammation and potentially restore immune balance in IDD. The dual role of IL-6 necessitates careful modulation to avoid adverse effects on tissue repair.	(93–95, 196, 197)
IL-17	Promotes recruitment and activation of neutrophils, contributing to chronic inflammation and ECM degradation	STAT3, RORγt, NF-κB, IL-23	IL-17 inhibitors (Secukinumab), RORγt inhibitors, IL-23 inhibitors (Ustekinumab), STAT3 inhibitors	IL-17 plays a significant role in chronic inflammation and ECM degradation in IDD. Targeting IL-17 and it’s signaling pathways could reduce inflammation and protect against tissue damage.	(19, 63, 73, 198, 199)
CXCL8 (IL-8)	Attracts neutrophils to the site of injury, contributing to acute and chronic inflammation in IDD.	NF-κB, MAPK, PI3K-Akt	CXCR1/CXCR2 inhibitors (Reparixin), NF-κB inhibitors, PI3K inhibitors, MAPK inhibitors	Targeting CXCL8 and its receptors could reduce neutrophil-driven inflammation and subsequent tissue damage in IDD. This approach could be particularly effective in the early stages of inflammation.Targeting specific cytokines and transcription factors involved in T cell differentiation (STAT3, FOXP3) could enhance therapeutic outcomes.	(64, 65, 67, 200, 201)
CCL2 (MCP-1)	Recruit monocytes and macrophages to the site of injury, contributing to chronic inflammation and tissue remodeling.	CCR2, NF-κB, MAPK	CCR2 antagonists (RS102895), NF-κB inhibitors, MAPK inhibitors	Inhibiting CCL2 could reduce the recruitment of pro-inflammatory macrophages and monocytes, potentially mitigating chronic inflammation and ECM degradation in IDD.	(33, 37, 38, 54, 202)

IL-1β, Interleukin 1 beta; TNF-α, Tumor Necrosis Factor alpha; MMPs, Matrix Metalloproteinases; NLRP3, NOD-like receptor pyrin domain-containing protein 3; ROS, Reactive Oxygen Species; NF-κB, Nuclear Factor kappa-light-chain-enhancer of activated B cells; MAPK, Mitogen-Activated Protein Kinase; Caspase-1, Cysteine-aspartic acid protease 1; JNK, c-Jun N-terminal kinase; AP-1, Activator Protein 1; Caspase-3, Cysteine-aspartic acid protease 3; IL-6, Interleukin 6; JAK, Janus Kinase; STAT3, Signal Transducer and Activator of Transcription 3; IL-17, Interleukin 17; RORγt, Retinoid-related orphan receptor gamma t; IL-23, Interleukin 23; CXCL8 (IL-8), C-X-C motif chemokine ligand 8 (also known as Interleukin 8); PI3K-Akt, Phosphatidylinositol 3-kinase/Protein kinase B pathway; CCL2 (MCP-1), C-C motif chemokine ligand 2 (also known as Monocyte Chemoattractant Protein-1); CCR2, C-C Chemokine Receptor Type 2.

#### 3.2.1 IL-1β

IL-1β is a key pro-inflammatory cytokine involved in IDD (4). It binds to its receptor IL-1R1 on the cell surface, activating downstream NF-κB and MAPK signaling pathways, thereby promoting the expression and activity of MMPs and accelerating the degradation of the disc matrix (83). Additionally, IL-1β induces oxidative stress and activates caspase signaling pathways, leading to the apoptosis of nucleus pulposus cells (NPCs) and annulus fibrosus cells (AFCs), further exacerbating disc degeneration (88).

#### 3.2.2 TNF-α

TNF-α is another pro-inflammatory cytokine that plays a significant role in IDD (89). It exerts its effects through TNFR1 and TNFR2 receptors, activating NF-κB and JNK signaling pathways, and regulating the expression of various inflammatory mediators (90). Recent studies indicate that TNF-α increases disc cell death by inducing oxidative stress and is positively correlated with the severity of disc degeneration (34, 91). Moreover, TNF-α promotes the expression of MMPs and ADAMTSs, accelerating matrix degradation (92).

#### 3.2.3 IL-6

IL-6 has a complex role in IDD. It binds to IL-6R and forms a complex with gp130, activating the JAK-STAT signaling pathway (93). As a pro-inflammatory cytokine, IL-6 promotes the expression of MMPs and ADAMTSs, accelerating matrix degradation (94). On the other hand, IL-6 is also considered to have anti-inflammatory effects, modulating immune cell responses through the STAT3 signaling pathway (95). This dual role makes IL-6’s function in disc degeneration complex and significant.

#### 3.2.4 Chemokines

Chemokines primarily regulate the migration and localization of immune cells during IDD. For example, CCL5 (RANTES) mediates its effects through the CCR5 receptor, attracting macrophages and neutrophils to the lesion site, enhancing local inflammatory responses (96). CXCL8 (IL-8) mediates its effects through CXCR1 and CXCR2 receptors, attracting neutrophils to the site of inflammation and promoting acute inflammatory responses. This cell migration not only exacerbates local tissue damage but also contributes to the maintenance of chronic inflammation (97).

### 3.3 Regulation of disc cell survival and apoptosis by inflammatory mediators

Inflammatory mediators regulate disc cell survival and apoptosis through various pathways. Pro-inflammatory cytokines such as IL-1β and TNF-α can activate several apoptosis-related signaling pathways, such as the caspase family, inducing cell apoptosis (98). Additionally, oxidative stress and mitochondrial dysfunction are important mechanisms by which inflammatory mediators induce cell death.

### 3.4 Regulation of matrix degradation

Pro-inflammatory cytokines like IL-1β and TNF-α can activate the caspase signaling pathway, inducing cell apoptosis. Furthermore, these cytokines can increase apoptosis rates by inducing oxidative stress and mitochondrial dysfunction (99). MMPs and ADAMTSs are major enzymes responsible for disc matrix degradation, and their expression and activity are regulated by various inflammatory factors. IL-1β and TNF-α can significantly increase the expression of these degradative enzymes by activating NF-κB and MAPK signaling pathways, thus accelerating matrix degradation. For example, IL-1β binds to its receptor IL-1R1 on the cell surface, activating downstream NF-κB and MAPK signaling pathways, promoting the expression of MMP-3 and MMP-13 genes and proteins, leading to the breakdown of collagen and proteoglycans (100). TNF-α exerts its effects through TNFR1 and TNFR2 receptors, activating NF-κB and JNK signaling pathways, regulating the expression of ADAMTS-4 and ADAMTS-5 genes and proteins, further promoting matrix degradation (34, 101).

### 3.5 Prospects for the application of novel inflammatory modulators

With a deeper understanding of the mechanisms of IDD, significant progress has been made in developing novel inflammatory modulators. For instance, IL-1 receptor antagonists (Anakinra) and TNF-α inhibitors (such as Etanercept) have shown promising results in preclinical studies (102, 103). Additionally, novel small molecule inhibitors targeting NF-κB and MAPK signaling pathways also show potential therapeutic prospects. These new drugs precisely modulate inflammatory responses, not only alleviating symptoms but potentially delaying or reversing the progression of disc degeneration fundamentally.

In summary, the mechanisms by which inflammatory cytokines function in IDD are complex and diverse. In-depth research into these mechanisms and regulatory pathways not only aids in understanding the pathophysiology of IDD but also provides an important theoretical basis for developing novel therapeutic strategies.

## 4 Signaling pathways of the immune system in intervertebral disc degeneration

### 4.1 NF-κB signaling pathway

The NF-κB signaling pathway plays a crucial role in disc degeneration and immune responses. NF-κB is a family of transcription factors that are key regulators of cellular stress and inflammatory responses (104). In IDD, NF-κB activation is primarily mediated by pro-inflammatory cytokines such as IL-1β and TNF-α (105). Upon binding to their receptors (IL-1R1 and TNFR1/TNFR2), these cytokines activate the IKK complex, leading to the phosphorylation and degradation of IκB proteins, thereby releasing NF-κB transcription factors. These factors then translocate to the nucleus and initiate the transcription of inflammatory genes (106). NF-κB activation leads to a cascade of downstream effects, including the upregulation of inflammatory cytokines (such as IL-6 and IL-8), chemokines (such as CCL5), and matrix metalloproteinases (MMP-3 and MMP-13) (107). These effects not only exacerbate the inflammatory response of disc cells but also accelerate matrix degradation (108). Moreover, NF-κB is linked to oxidative stress, mitochondrial dysfunction, and ferroptosis, which further promote cellular damage and matrix degradation (109). NF-κB also influences cell death pathways, including ferroptosis, cuproptosis, and pyroptosis, by regulating Bcl-2 family proteins (110). Given the pivotal role of NF-κB in disc degeneration, targeting this pathway presents significant therapeutic potential. Currently, some NF-κB inhibitors have shown promising results in preclinical studies. For instance, IKKβ inhibitors significantly slowed the progression of disc degeneration in animal models (111). Additionally, small-molecule drugs targeting the NF-κB pathway, such as BAY 11–7082 and Parthenolide, have demonstrated potential in inhibiting disc degeneration-related inflammatory responses (112).

### 4.2 MAPK signaling pathway

The activation of the MAPK signaling pathway in disc cells and its impact on immune responses have also garnered significant attention. The MAPK family includes three major pathways: ERK, JNK, and p38, which play critical roles in cell proliferation, differentiation, stress response, and death (103). In IDD, the MAPK signaling pathway is also activated by inflammatory cytokines such as IL-1β and TNF-α, influencing disc cell functions through various mechanisms (113). The ERK pathway is primarily involved in cell proliferation and differentiation, but its excessive activation in an inflammatory environment may lead to abnormal cell proliferation and matrix metabolic imbalance (114). The JNK and p38 pathways are mainly involved in stress responses, with their activation leading to increased expression of pro-inflammatory genes and promoting novel cell death pathways such as ferroptosis and cuproptosis by regulating caspase family proteins (115). Additionally, the MAPK signaling pathway is involved in epigenetic and post-transcriptional regulation, affecting gene expression and protein function by modulating histone modifications and RNA stability. Regulators targeting the MAPK pathway hold broad prospects in the treatment of disc degeneration (116). For example, the p38 MAPK inhibitor SB203580 has shown efficacy in suppressing inflammatory responses in disc cells *in vitro*, and the JNK inhibitor SP600125 has demonstrated potential in slowing disc degeneration in animal models (117).

### 4.3 JAK-STAT signaling pathway

The JAK-STAT pathway mediates signal transduction through cytokine receptors and plays a vital role in inflammation and immune responses. Cytokines such as IL-6 and IL-10 bind to their receptors, activating JAK kinases, which in turn activate STAT transcription factors. These STAT factors then translocate to the nucleus to regulate the expression of target genes (118). In IDD, the JAK-STAT signaling pathway contributes to disease progression by regulating the expression of inflammatory cytokines and the activation of immune cells. For example, IL-6 activates the JAK-STAT3 pathway, promoting the expression of pro-inflammatory genes, thereby exacerbating inflammation and matrix degradation in the disc (93). The JAK-STAT signaling pathway is also closely related to disc cell survival and death, influencing cell death pathways such as ferroptosis, cuproptosis, and pyroptosis by regulating the expression of Bcl-2 family proteins (119). Recent studies have also found that the JAK-STAT pathway plays an important role in epigenetic regulation, affecting gene expression through DNA methylation and histone modifications. JAK-STAT inhibitors show promising potential in the treatment of disc degeneration. For instance, Tofacitinib, a JAK inhibitor, has demonstrated efficacy in suppressing disc degeneration-related inflammatory responses in preclinical studies (120). Additionally, small-molecule inhibitors targeting STAT3 have shown potential in regulating inflammatory responses and slowing the degeneration process (121).

### 4.4 NLRP3 inflammasome

The NLRP3 inflammasome is a multiprotein complex that senses intracellular danger signals, activating caspase-1, which promotes the maturation and release of IL-1β and IL-18 (122). In IDD, danger signals such as oxidative stress, mitochondrial damage, and matrix degradation products can activate the NLRP3 inflammasome. Activation of the NLRP3 inflammasome leads to a strong local inflammatory response and cell death in the disc (34). The release of IL-1β not only promotes the expression of more inflammatory cytokines but also further activates the NF-κB and MAPK signaling pathways through a positive feedback mechanism, exacerbating disc degeneration. NLRP3 is also closely related to pyroptosis, an inflammatory form of cell death, which further intensifies the local inflammatory response (123). Activation of the NLRP3 inflammasome also involves ubiquitination processes that regulate protein degradation and function (124). The potential of NLRP3 inflammasome inhibitors in the treatment of disc degeneration is gradually becoming apparent. For example, MCC950, a specific NLRP3 inhibitor, significantly slowed the progression of disc degeneration in animal models (125). Additionally, other NLRP3 inhibitors such as Bavachin (BHB) and Berberine have shown efficacy in suppressing inflammatory responses and protecting the disc in preclinical studies (126).

**Figure 2:**
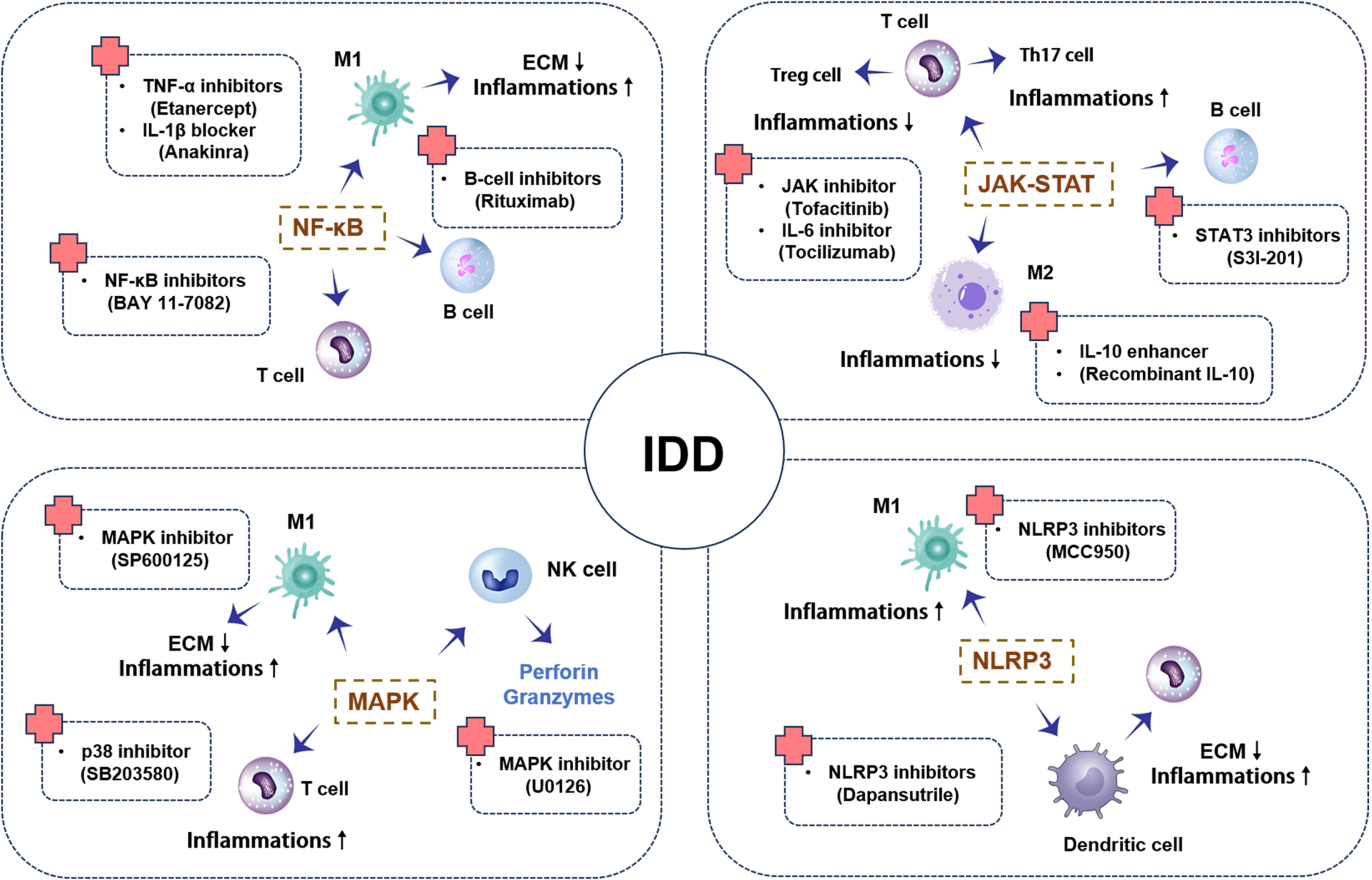
Schematic representation of key signaling pathways in intervertebral disc degeneration: This diagram depicts the activation and interactions of key signaling pathways, including NF-κB, MAPK, JAK-STAT, and NLRP3, during IDD. It includes pro-inflammatory actions, anti-inflammatory effects, and apoptosis. The schematic shows how these signaling pathways transmit signals between different immune cells, triggering inflammatory responses that further promote or inhibit the progression of disc degeneration.

In summary, signaling pathways such as NF-κB, MAPK, JAK-STAT, and NLRP3 inflammasome play critical roles in disc degeneration (**Table 3**). In-depth studies of the activation mechanisms and functions of these pathways not only aid in understanding the pathophysiological processes of disc degeneration but also provide an important theoretical basis for developing novel therapeutic strategies (**Figure 2**). Increasing evidence highlights the complex crosstalk among these signaling pathways. For instance, NF-κB activation primes the expression of NLRP3 inflammasome components, thus facilitating pyroptosis under stress (127). Meanwhile, IL-6-mediated activation of the JAK-STAT3 pathway can modulate NF-κB transcriptional output, amplifying inflammation (128). MAPK pathways (notably p38 and JNK) intersect with both NF-κB and NLRP3 activation cascades, creating a positive feedback loop (129). This synergy suggests that dual or multi-pathway inhibitors—such as those targeting both NF-κB and NLRP3—may offer enhanced therapeutic efficacy. Notably, intradiscal AAV-CRISPR/Cas9 knock-down of β-catenin not only preserves notochord-derived cells and annulus integrity but also attenuates NF-κB-driven NLRP3 priming, highlighting β-catenin as an upstream modulator of this inflammasome axis and positioning gene editing as a precise, multi-pathway regenerative strategy (130).

**Table 3:** Signaling pathways in IDD and associated therapeutic interventions.

Signaling pathway	Role in IDD	Key regulatory molecules	Potential targets and drugs	Clinical implications	References
NF-κB Pathway	Central mediator of inflammation and ECM degradation in IDD. Activated by pro-inflammatory cytokines (IL-1β, TNF-α) leading to the transcription of inflammatory genes and degradative enzymes.	IKK complex, IκB, p65/p50, IL-6, IL-8, MMPs, ROS	IKK inhibitors (BAY 11-7082), NF-κB inhibitors (Parthenolide), ROS scavengers (N-acetylcysteine)	Targeting the NF-κB pathway can significantly reduce inflammation and ECM degradation in IDD. Inhibiting this pathway may prevent chronic inflammation and protect against progressive disc degeneration.	(104–106, 203, 204)
MAPK Pathway	Regulates cellular responses to stress and inflammation. In IDD, it promotes the expression of pro-inflammatory cytokines and enzymes involved in ECM degradation.	ERK, JNK, p38, MMPs, AP-1, IL-1β, TNF-α	p38 MAPK inhibitors (SB203580), JNK inhibitors (SP600125), ERK inhibitors, AP-1 inhibitors	Inhibiting the MAPK pathway can mitigate the inflammatory response and protect against ECM degradation in IDD. Targeting specific MAPK components may allow for more precise therapeutic interventions.	(103, 113, 114, 117, 205, 206)
JAK-STAT Pathway	Mediates cytokine signaling involved in inflammation and immune responses. In IDD, it promotes inflammatory gene expression and contributes to chronic inflammation and disc cell apoptosis.	JAK1/2/3, STAT3/5, IL-6, IL-10, SOCS3	JAK inhibitors (Tofacitinib), STAT3 inhibitors (WP1066), SOCS3 mimetics, IL-6 inhibitors (Tocilizumab)	Targeting the JAK-STAT pathway could reduce inflammation and apoptosis in IDD, potentially offering a strategy to halt or reverse disease progression. Combination therapies targeting both JAK and STAT proteins may provide enhanced efficacy.	(118–120, 207, 208)
NLRP3 Inflammasome	Activates caspase-1, leading to the maturation of IL-1β and IL-18, promoting inflammation and pyroptosis in IDD.	NLRP3, ASC, Caspase-1, IL-1β, IL-18, ROS	NLRP3 inhibitors (MCC950), Caspase-1 inhibitors (Ac-YVAD-cmk), ROS scavengers, IL-1β inhibitors (Anakinra)	Targeting NLRP3 inflammasome can reduce inflammation and cell death in IDD, potentially preventing further damage. Combining NLRP3 inhibitors with antioxidants may improve outcomes.	(88, 122–124, 209, 210)

NF-κB, Nuclear Factor kappa-light-chain-enhancer of activated B cells; IKK, IκB kinase; IκB, Inhibitor of kappa B; IL-6, Interleukin 6; MMPs, Matrix Metalloproteinases; ROS, Reactive Oxygen Species; MAPK, Mitogen-Activated Protein Kinase; ERK, Extracellular Signal-Regulated Kinase; JNK, c-Jun N-terminal kinase; AP-1, Activator Protein 1; IL-1β, Interleukin 1 beta; TNF-α, Tumor Necrosis Factor alpha; JAK, Janus Kinase; STAT3, Signal Transducer and Activator of Transcription 3; SOCS3, Suppressor of Cytokine Signaling 3; NLRP3, NOD-like receptor pyrin domain-containing protein 3; ASC, Apoptosis-associated Speck-like Protein Containing a CARD; Caspase-1, Cysteine-aspartic acid protease 1; IL-18, Interleukin 18.

## 5 The relationship between the immune microenvironment and ECM degradation

### 5.1 Role of inflammatory mediators in ECM degradation

The ECM, mainly composed of collagen, proteoglycans, and other matrix molecules, provides structural support and elasticity to the disc. Inflammatory mediators such as IL-1β and TNF-α significantly accelerate ECM degradation by upregulating the expression of MMPs and ADAMTS proteases (131). These inflammatory cytokines not only directly promote ECM degradation but also exacerbate the degenerative process by inducing mitochondrial autophagy in nucleus pulposus cells and altering the balance of ECM synthesis and degradation (132).

### 5.2 Role of miRNAs and lncRNAs in regulating inflammatory mediators and ECM degradation

During IDD, non-coding RNAs such as microRNAs (miRNAs) and long non-coding RNAs (lncRNAs) play increasingly recognized roles in regulating inflammatory mediators and ECM degradation. For example, miR-27b inhibits MMP-13 expression by directly targeting its 3’UTR region, thereby slowing ECM degradation (133). Studies indicate that miRNAs can regulate the expression of MMPs and ADAMTS through various pathways, influencing ECM degradation and the progression of disc degeneration. Furthermore, lncRNA HOTAIR, by binding to polycomb protein EZH2, promotes H3K27me3 modification, thereby inhibiting the expression of MMP-1 and MMP-3 and reducing matrix degradation (134). These molecular mechanisms illustrate the multi-layered regulatory roles of non-coding RNAs in ECM degradation. Additionally, miR-21 indirectly promotes the expression of MMP-3 and MMP-9 by downregulating tissue inhibitor of metalloproteinases 3 (TIMP3), leading to accelerated ECM degradation (135). LncRNA MEG3, through interaction with the tumor suppressor p53, inhibits the expression of MMP-2 and MMP-9, protecting the ECM from excessive degradation (136). Notably, non-coding RNAs not only play important roles in gene expression regulation but also influence signal transduction and intercellular communication, playing key roles in the pathogenesis of disc degeneration. For instance, lncRNA TUG1 regulates the Wnt/β-catenin signaling pathway, affecting inflammatory responses and ECM degradation (137). Future research should further elucidate the specific functions and mechanisms of non-coding RNAs in disc degeneration and explore their potential as therapeutic targets.

### 5.3 Immunoregulatory role of ECM degradation products

ECM degradation not only impacts the structure and function of the disc but also produces degradation products that have important immunoregulatory roles. ECM degradation products such as collagen fragments, proteoglycan fragments, and hyaluronic acid fragments can act as damage-associated molecular patterns (DAMPs), activating immune cells and triggering and sustaining inflammatory responses. For example, collagen fragments activate downstream NF-κB and MAPK signaling pathways through TLR2 and TLR4, inducing the expression of IL-1β, TNF-α, and IL-6, further exacerbating inflammation and ECM degradation (138). Hyaluronic acid fragments can similarly activate macrophages through the same pathways, enhancing their pro-inflammatory responses (55). Additionally, proteoglycan fragments such as aggrecan and versican fragments, by binding to the CD44 receptor, activate macrophages and dendritic cells, enhancing their pro-inflammatory and antigen-presenting functions (139).

Despite significant progress in the study of IDD and ECM degradation, many gaps and controversies remain. First, the molecular mechanisms of ECM degradation are not yet fully elucidated. Although many key proteases and signaling pathways have been identified, their specific roles and interactions at different pathological stages require further investigation. Additionally, the precise mechanisms by which inflammatory mediators and ECM degradation products regulate the immune microenvironment, especially in different types of immune cells (such as macrophages and T cells), remain unclear. Moreover, current animal models and *in vitro* systems have limitations in fully simulating the complex pathological processes of human IDD. Many findings have yet to be validated in clinical trials, and effective strategies for clinical treatment are still facing challenges. Therefore, future research needs to develop more precise and effective experimental models and adopt multidisciplinary approaches to further uncover the complex mechanisms of IDD. In conclusion, although progress has been made in understanding the relationship between IDD and ECM degradation, many scientific questions and technical challenges remain to be addressed to advance the field and provide more effective strategies for clinical treatment.

## 6 Potential of immune regulation in the treatment of intervertebral disc degeneration

### 6.1 Anti-inflammatory drugs

In recent years, the application of anti-inflammatory drugs in IDD has increased. Non-steroidal anti-inflammatory drugs (NSAIDs) are the most commonly used anti-inflammatory drugs, reducing prostaglandin synthesis by inhibiting cyclooxygenase (COX) to alleviate pain and inflammation. However, long-term use of NSAIDs can cause gastrointestinal and cardiovascular side effects (140). More targeted anti-inflammatory drugs, such as selective COX-2 inhibitors, TNF-α antagonists, and IL-1β inhibitors, have shown better therapeutic effects and fewer side effects. For example, studies have shown that the use of TNF-α antagonists (such as Etanercept) can effectively reduce pain and inflammation in patients with IDD (141). Additionally, IL-1β inhibitors (such as Anakinra) block the IL-1β signaling pathway, slowing the progression of IDD and improving patient symptoms (142). These novel anti-inflammatory drugs offer new directions for the treatment of IDD, but their long-term efficacy and safety need further study.

### 6.2 Biologics

Biologics also show great potential in the treatment of IDD. Biologics are protein-based drugs produced through genetic engineering techniques, capable of precisely targeting specific inflammatory factors or cells. Studies have shown that using monoclonal antibodies to block specific inflammatory factors can significantly slow the progression of IDD. For instance, anti-IL-6 receptor monoclonal antibody (such as Tocilizumab) has shown good anti-inflammatory effects in animal models, reducing disc tissue damage and inflammatory cell infiltration (143). Furthermore, stem cell therapy, as an emerging biological treatment, has shown potential in regenerating and repairing disc tissue (24, 101). Mesenchymal stem cells (MSCs) can regulate immune responses and promote disc cell proliferation and ECM synthesis by secreting various anti-inflammatory factors and growth factors (144). Clinical trials have shown that MSC injections can significantly improve symptoms in patients with IDD and promote disc tissue repair (145, 146). Despite their promise, MSC therapies face challenges in clinical application. The harsh, hypoxic, and nutrient-poor environment of the degenerated disc significantly limits MSC survival and function (147). Additionally, unmodified MSCs may have limited homing ability and paracrine activity. Recent advances, such as engineering MSCs with hypoxia-adaptive genes or embedding them in biomaterial scaffolds, have shown improved outcomes (148). Exosome-based delivery also emerges as a minimally immunogenic and stable alternative (145, 149).

### 6.3 Immunomodulatory therapy

Immunomodulatory therapy aims to modulate the immune system’s function, restore immune balance, and slow or reverse the progression of IDD. Recent studies on regulatory T cells (Tregs) and macrophages have shown significant therapeutic potential. Research indicates that Tregs can secrete anti-inflammatory cytokines (such as IL-10 and TGF-β), suppressing inflammatory responses and slowing IDD progression (150, 151). Additionally, the role of macrophages in IDD has gained considerable attention. M1 macrophages have pro-inflammatory effects, while M2 macrophages have anti-inflammatory and tissue repair functions. Promoting the polarization from M1 to M2 macrophages can significantly slow the progression of IDD (152, 153). An emerging immunomodulatory therapy involves using exosomes, which are nanoscale particles capable of delivering anti-inflammatory and reparative signals (**Table 4**). Studies have shown that exosomes derived from MSCs can modulate immune responses and slow disc degeneration (24).

**Table 4:** Immune regulation strategies in the treatment of IDD.

Immune modulation strategy	Role in IDD	Mechanism of action	Potential therapies	Clinical implications	References
Macrophage Polarization	Modulates inflammation and tissue repair through the balance of M1 (pro-inflammatory) and M2 (anti-inflammatory) macrophages.	M1 macrophages secrete pro-inflammatory cytokines (IL-1β, TNF-α) that promote ECM degradation, while M2 macrophages secrete anti-inflammatory cytokines (IL-10, TGF-β) that promote tissue repair.	Agents promoting M2 polarization (IL-4, IL-13, PPARγ agonists), NLRP3 inhibitors (MCC950)	Targeting macrophage polarization could reduce chronic inflammation and promote tissue repair in IDD, potentially slowing disease progression. Modulating the M1/M2 balance is crucial for achieving optimal therapeutic outcomes.	(88, 211–213)
Cytokine Inhibition	Reduces chronic inflammation and tissue damage by blocking key pro-inflammatory cytokines.	Inhibiting cytokines like TNF-α, IL-1β, and IL-17 disrupts their signaling pathways, reducing the production of inflammatory mediators and ECM-degrading enzymes.	TNF-α inhibitors (Etanercept), IL-1β inhibitors (Anakinra), IL-17 inhibitors (Secukinumab)	Inhibiting pro-inflammatory cytokines can help control chronic inflammation and prevent further disc degeneration. This approach is particularly effective in reducing pain and slowing disease progression in IDD.	(142, 179, 214–216)
Regulatory T Cells (Tregs)	Suppress excessive immune responses and promote an anti-inflammatory environment in IDD.	Tregs secrete anti-inflammatory cytokines (IL-10, TGF-β) and inhibit pro-inflammatory T cells, reducing overall inflammation and tissue damage.	Treg enhancers (Low-dose IL-2), TGF-β therapy, FOXP3 gene therapy	Enhancing Treg activity could suppress chronic inflammation and autoimmunity in IDD, potentially leading to improved tissue repair and reduced disease progression	(217–219)
Inflammasome Inhibition	Reduces inflammation and cell death by targeting the inflammasome pathway.	Inhibiting NLRP3 inflammasome activation reduces the production of IL-1β and IL-18, key mediators of inflammation and pyroptosis in IDD.	NLRP3 inhibitors (MCC950) Caspase-1 inhibitors (VX-765) ROS scavengers	Inhibiting the inflammasome pathway can significantly reduce inflammation and prevent cell death in IDD, potentially slowing or reversing disc degeneration.	(220–222)
MSC Therapy	MSCs can differentiate into disc cells and modulate the immune environment by secreting anti-inflammatory factors.	MSCs secrete factors that promote repair, reduce inflammation, and modulate immune cells. They can also differentiate into nucleus pulposus-like cells to regenerate disc tissue.	MSC transplantation, MSC-derived exosomes, Combination therapies	MSC therapy offers a regenerative approach to treating IDD by promoting tissue repair and modulating the immune environment. This strategy has the potential to not only halt disease progression but also restore disc function.	(24, 144, 223, 224)

M1, Pro-inflammatory macrophages (Type 1 macrophages); M2, Anti-inflammatory macrophages (Type 2 macrophages); IL-1β, Interleukin 1 beta; TNF-α, Tumor Necrosis Factor alpha; ECM, Extracellular Matrix; TGF-β, Transforming Growth Factor beta; IL-10, Interleukin 10; PPARγ, Peroxisome Proliferator-Activated Receptor gamma; NLRP3, NOD-like receptor pyrin domain-containing protein 3; IL-4, Interleukin 4; IL-13, Interleukin 13; Tregs, Regulatory T cells; FOXP3, Forkhead box P3; MSC, Mesenchymal Stem Cell; NLRP3, NOD-like receptor pyrin domain-containing protein 3; ROS, Reactive Oxygen.

## 7 Future research directions and challenges

### 7.1 Discovery of novel immune biomarkers

Identifying and validating new immune biomarkers is crucial for better understanding and monitoring the immune environment in IDD. Recent studies have emphasized the role of inflammatory cytokines like IL-6 and TNF-α, which are not only markers of inflammation but also active contributors to disc degeneration. For example, a 2021 study highlighted how elevated IL-6 and TNF-α levels in disc tissue correlate with increased MMP activity, leading to accelerated ECM degradation (154).

Beyond these traditional markers, emerging research has identified specific microRNAs (miRNAs) and long non-coding RNAs (lncRNAs) as promising biomarkers in IDD (144) (150, 155). Notably, miR-146a and miR-21 have been shown to regulate inflammatory responses and ECM homeostasis, with studies in 2022 and 2023 respectively demonstrating their potential as therapeutic targets in IDD (156). Additionally, lncRNAs like HOTAIR and MEG3 have been implicated in disc degeneration, with HOTAIR promoting ECM degradation and apoptosis, while MEG3 offers protective effects by inhibiting MMP expression (157). The discovery of these markers has been significantly advanced by high-throughput screening and systems biology approaches, such as single-cell RNA sequencing (scRNA-seq), which have enabled the identification of distinct immune cell subsets and molecular networks within degenerated disc tissues (21, 158). A 2022 study using scRNA-seq, for example, revealed distinct macrophage populations that play differential roles in inflammation and tissue repair (68). Furthermore, systems biology approaches integrating multi-omics data have provided comprehensive insights into the molecular landscape of IDD, as evidenced by a 2023 study that uncovered key regulatory networks involving miRNAs, lncRNAs, and cytokines (159). These advancements underscore the potential of novel biomarkers to improve early detection, monitor disease progression, and guide personalized treatment strategies in IDD.

### 7.2 Development of personalized immunotherapy

Developing personalized immunotherapy strategies tailored to patients’ unique immune profiles is a promising future direction in the treatment of IDD. For example, analyzing patients’ immune cell lineages and inflammatory cytokine levels can help design targeted treatment plans, such as using specific antibodies or small-molecule inhibitors to block key inflammatory pathways (160). Recent advancements have highlighted the potential of specific immune modulators and gene therapies to achieve precision medicine. By analyzing individual immune cell lineages and inflammatory cytokine levels, clinicians can design targeted treatment plans that are more effective and have fewer side effects. For example, a 2021 study demonstrated that patients with elevated Th17 cell levels responded favorably to IL-17 inhibitors, emphasizing the importance of immune profiling in therapy selection (161). Additionally, modulating macrophage polarization has shown potential, with research indicating that adjusting the M1/M2 balance through specific small-molecule inhibitors can reduce chronic inflammation and promote tissue repair in degenerative discs. These approaches underscore the value of personalized immunotherapy in addressing the specific immune dysfunctions associated with IDD (51).

Gene editing technologies, particularly CRISPR/Cas9, are at the forefront of personalized immunotherapy, offering precise modifications of genes involved in inflammatory responses. Recent studies have explored the use of CRISPR/Cas9 to edit genes within the NF-κB pathway, leading to reduced inflammation and slowed disease progression in IDD models (130). Moreover, correcting mutations responsible for excessive cytokine production has shown promise in reversing some of the pathological changes associated with the disease. The integration of multi-omics data, including genomics, transcriptomics, and proteomics, further enhances the development of personalized treatments by providing a comprehensive understanding of each patient’s unique disease biology. This data-driven approach enables the identification of key biomarkers and therapeutic targets, paving the way for more tailored and effective therapies. As personalized immunotherapy continues to evolve, future directions may include combination therapies that integrate multiple targeted approaches, potentially revolutionizing the management of IDD and improving patient outcomes.

### 7.3 Multidisciplinary collaboration

Promoting multidisciplinary collaboration is essential for advancing the understanding and treatment of IDD. The complexity of IDD, involving mechanical, biochemical, and immunological factors, necessitates an integrated approach drawing on expertise from fields such as immunology, molecular biology, bioengineering, and clinical medicine. By fostering collaboration across these disciplines, researchers can develop innovative technologies and therapeutic strategies that address the multifaceted nature of IDD. For example, bioengineering plays a crucial role in designing advanced biomaterials and drug delivery systems to enhance the therapeutic effects of immunomodulators. A 2021 study demonstrated the use of a novel hydrogel-based scaffold for the sustained release of anti-inflammatory drugs directly into the degenerated disc, significantly reducing local inflammation and promoting tissue regeneration (15, 162). These innovations highlight the importance of combining materials science, pharmacology, and clinical expertise to create biocompatible and effective treatments.

Moreover, integrating molecular biology with clinical research can accelerate the translation of basic science discoveries into therapeutic interventions. Identifying specific molecular targets, such as cytokines or signaling pathways involved in IDD, requires a deep understanding of both the underlying biology and the clinical manifestations of the disease. Collaborative efforts between molecular biologists and clinicians have led to the discovery of novel biomarkers for early-stage IDD, paving the way for personalized treatment plans. Additionally, collaboration between engineering and clinical medicine has the potential to revolutionize IDD treatment through the creation of advanced medical devices and surgical techniques. For instance, recent advancements in 3D bioprinting technology have enabled the development of anatomically accurate disc implants customized to fit individual patient anatomies, offering a promising alternative to traditional surgical interventions (163). Such breakthroughs are made possible through the joint efforts of engineers, biologists, and clinicians, who together bridge the gap between experimental technology and practical application in patient care.

### 7.4 Unresolved questions

The specific functions and regulatory mechanisms of immune cells in IDD remain incompletely understood, posing significant challenges to the development of targeted therapies. While existing studies have established that immune cells such as macrophages and T cells play pivotal roles in the progression of IDD, the precise mechanisms by which these cells contribute to the disease are still under investigation. For instance, macrophages, which can polarize into pro-inflammatory M1 and anti-inflammatory M2 phenotypes, exhibit dynamic and context-dependent behavior in IDD. A 2022 study by Zhang et al. indicated that M1 macrophages are predominant in the early stages of disc degeneration, contributing to tissue damage through the secretion of pro-inflammatory cytokines and matrix-degrading enzymes (50). However, the transition from M1 to M2 macrophages, which theoretically should promote tissue repair and resolution of inflammation, does not appear to occur effectively in many cases of chronic IDD (164). This suggests a potential dysregulation in macrophage polarization, which could be a critical factor in the persistence of inflammation and progression of the disease.

Moreover, the interaction networks between different immune cells, such as macrophages, T cells, and other immune components, are not yet fully mapped out. T cells, particularly the balance between pro-inflammatory Th1/Th17 cells and anti-inflammatory Treg cells, play a crucial role in modulating the immune environment within the degenerative disc (165). However, recent studies suggest that this balance is often disrupted in IDD, leading to a chronic inflammatory state that exacerbates tissue degradation. For example, a 2021 study highlighted that increased Th17 cell activity correlates with more severe disc degeneration, while Treg cell dysfunction may contribute to the failure of inflammation resolution (160). The specific molecular signals and pathways that govern these immune cell interactions in the disc microenvironment are still poorly understood, and unraveling these mechanisms could reveal new therapeutic targets. Additionally, the role of other immune cells, such as dendritic cells and B cells, in IDD remains underexplored. These cells could contribute to the chronicity of inflammation or interact with resident disc cells in ways that influence disease outcomes. In-depth research into these unresolved questions is essential for advancing our understanding of IDD pathophysiology and for the identification of novel, more effective therapeutic strategies.

### 7.5 Technical bottlenecks

Current research faces several technical challenges. For example, existing animal models cannot fully simulate the complex pathological processes of human IDD, limiting the extrapolation of research results (166). Gene editing approaches such as CRISPR/Cas9 offer precise modulation of inflammatory pathways. However, off-target mutations, immunogenicity of the delivery vector, and ethical concerns remain significant barriers to clinical adoption (167, 168). Strategies like high-fidelity Cas9 variants, exosome-based delivery systems, and transient editing protocols are being explored to mitigate these risks (169). Additionally, obtaining human samples poses certain difficulties, affecting the conduct of large-scale studies. To overcome these challenges, more advanced animal models and *in vitro* experimental systems need to be developed, utilizing high-throughput screening techniques to discover new immune regulatory factors.

### 7.6 Overcoming challenges

Recent research directions and technological advances provide new ideas and methods for advancing the study of interactions between IDD and the immune system. For example, single-cell RNA sequencing technology can accurately analyze different cell types and their interactions within disc tissue, helping understand immune cells’ functions at different pathological stages (170, 171). Additionally, the application of organoid technology is gradually emerging, allowing better simulation of IDD’s pathological processes *in vitro*, and screening potential therapeutic drugs (172). Furthermore, the application of artificial intelligence (AI) and machine learning (ML) technologies in biomedical research provides new tools for IDD research. These technologies can help analyze large datasets of genes, proteins, and metabolites, discovering new disease biomarkers and therapeutic targets (173). International cooperation and data-sharing platforms also help overcome sample acquisition limitations, promoting research progress through shared resources and technologies.

IDD is a prevalent condition marked by the progressive deterioration of disc structure and function. This review explores the pathophysiological mechanisms, emphasizing the role of ECM degradation, immune responses, and key signaling pathways like NF-κB, MAPK, and JAK-STAT. Advances in understanding immune microenvironments have highlighted potential therapeutic targets, including anti-inflammatory drugs, biologics, and immunomodulatory therapies. Future research should focus on identifying novel immune biomarkers, developing personalized treatments, and fostering multidisciplinary collaboration to enhance therapeutic strategies and improve patient outcomes.
